# Klinisch untypischer Verlauf eines frisch perforierten Hornhautulkus

**DOI:** 10.1007/s00347-021-01381-w

**Published:** 2021-04-19

**Authors:** E. Sinicin, D. Brockmann, M. Bartram, C. Framme, A. Bajor

**Affiliations:** Universitätsklinik für Augenheilkunde, Medizinische Hochschule Hannover, Carl-Neuberg-Str. 1, 30625 Hannover, Deutschland

## Anamnese

**Figure 1:**
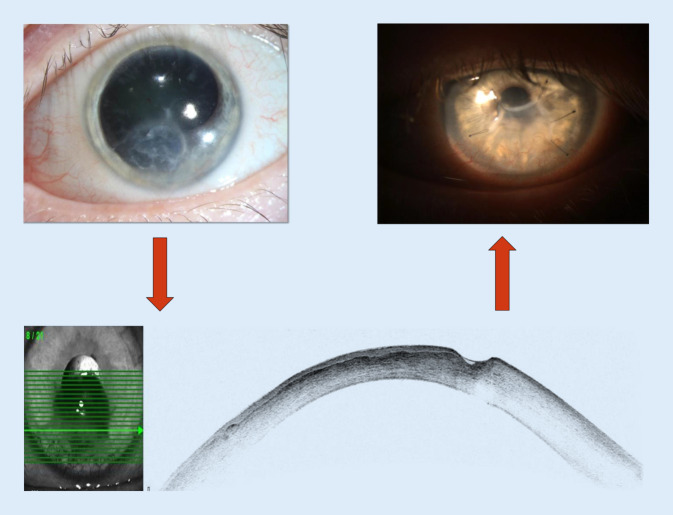

Ein 48-jähriger Patient mit langjährig bekannter Hornhautproblematik wurde als Notfall mit der Diagnose eines perforierten Hornhautulkus und bestehender Autotamponade des linken Auges vom Facharzt eingewiesen. Anamnestisch bestand eine linksseitige schmerzlose Sehminderung seit dem Vortag. Der Patient war in gutem Allgemeinzustand. Als Folge einer Fazialislähmung, welche seit der operativen Entfernung eines juvenilen Angiofibroms bestand, hatte er bereits eine Sicca-Symptomatik mit rezidivierenden Hornhauterosionen, eine Durchwanderungskeratitis sowie mehrere Herpeskeratitiden am betroffenen Auge erlitten. Deshalb waren mehrfach intensivierte antibiotische und antimykotische Lokaltherapien sowie mehrere Amniontransplantationen notwendig geworden. Im Oktober des vorherigen Jahres erfolgte bei fortgeschrittener Katarakt und hinteren Synechien eine Phakoemulsifikation mit Hinterkammerlinsenimplantation. Mehrere Wochen nach der Operation (4 Monate vor der Notfallvorstellung) wurde der Patient aufgrund eines Hornhautulkus bei uns vorstellig (Abb. 1 links oben). Es wurde eine intensivierte antibiotische Lokaltherapie mit Kanamycin- und Moxifloxacin-Augentropfen und Ganciclovir-Augengel begonnen. Bei der Verlaufskontrolle 2 Monate später zeigte sich im Vorderabschnitts-OCT neben einer Zunahme der zentralen Hornhautverdünnung auch eine inferiore Verdünnung (Abb. 1 unten), sodass eine Amnionmembranaufnähung erfolgte. Der Patient wurde 14 Tage vor Notfallvorstellung zur Befundkontrolle vorstellig. Zu diesem Zeitpunkt wies das Auge eine geschlossene Hornhaut sowie einen klaren Funduseinblick ohne Glaskörperreiz oder -infiltration auf (Abb. 1 rechts oben). Die weitere Anamnese bezüglich Allgemeinerkrankungen und Allergien war bis auf eine Epilepsie infolge eines Angiofibroms unauffällig.

## Klinischer Befund

Der Patient erkannte bei Aufnahme am betroffenen linken Auge Lichtschein mit defekter Projektion. Der Bulbus war weich. Es zeigten sich eine leichte Injektion der Bindehaut und ein zentral perforiertes Hornhautulkus mit Irisinkarzeration. Die peripher stehende Vorderkammer zeigte eine milde Fibrinreaktion ohne Hypopyon. Ein Einblick auf tiefer liegende Strukturen war nicht möglich.

## Therapie und Verlauf

In Zusammenschau der Historie und des Befundes am linken Auge wurde bei Diagnose eines perforierten Hornhautulkus mit Irisinkarzeration eine Keratoplastik (KPL) à chaud indiziert.

Präoperativ wurden neben dem Einsetzen einer Kontaktlinse zur Stabilisierung des Augentonus eine prophylaktische Lokaltherapie mit Moxifloxacin und Kanamycin sowie die intravenöse Therapie mit Flucloxacillin und Ceftriaxon eingeleitet. Laborchemisch zeigte sich eine leichte Erhöhung des C‑reaktiven Proteins (CRP) mit 6,2 mg/l sowie der Leukozyten mit 12.000/µl.

**Figure 2:**
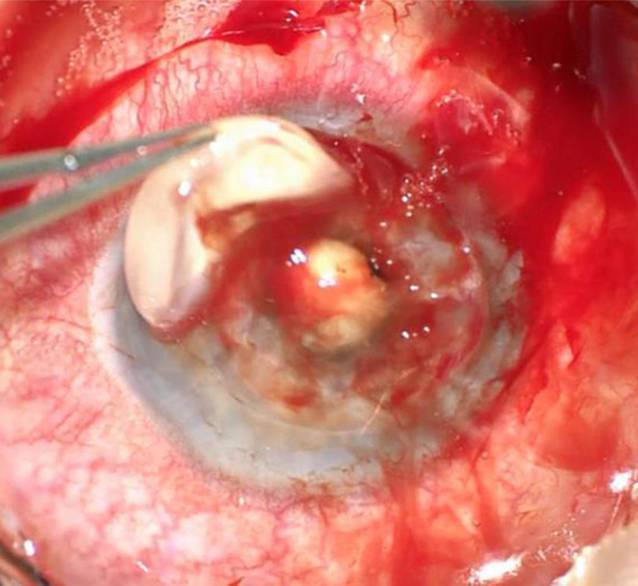

**Figure 3:**
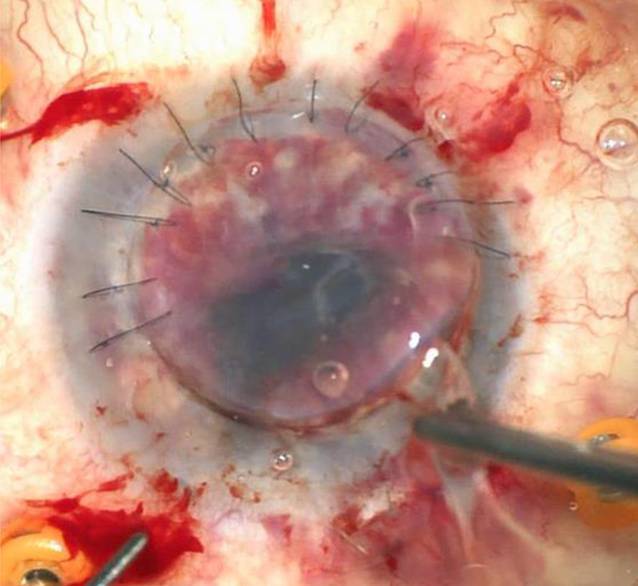

**Figure 4:**
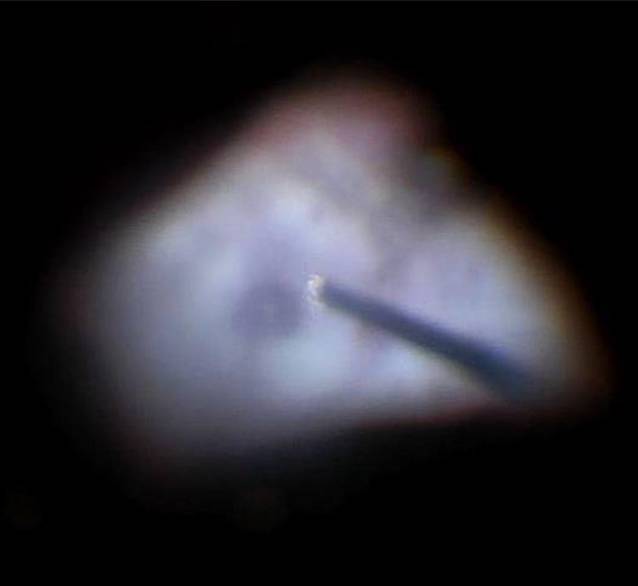

**Figure 5:**
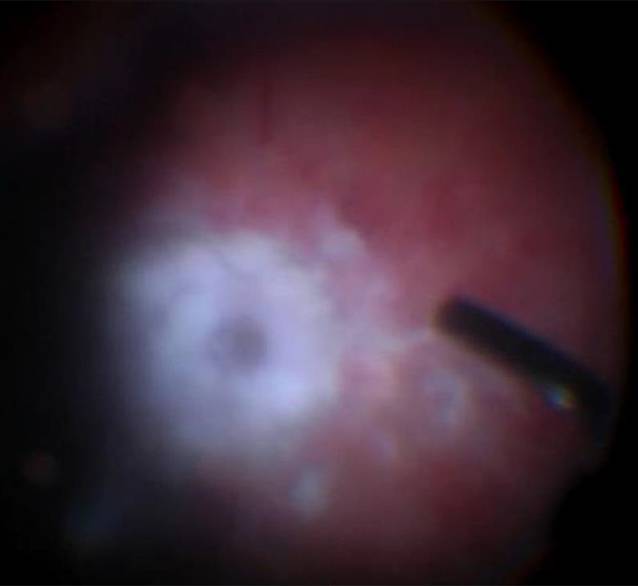

Intraoperativ wurde die KPL à chaud (Abb. 2) durch eine ausgeprägte Vis-à-tergo erschwert, und die Vorderkammer ließ sich zunächst bei Seclusio pupillae nicht stellen. Es wurde eine Iridektomie angelegt, aus der überraschenderweise Pus austrat. Dieser deutete auf eine tiefe Infektion des Augapfels hin. Der Eingriff wurde um eine IOL-Explantation (Abb. 3) und Pars-plana-Vitrektomie erweitert. Nach Entfernung der IOL zeigte sich eine fulminante Endophthalmitis mit massiv infiltriertem Glaskörper, multiplen schneeweißen, der Netzhaut aufliegenden Membranen, retinalen Infiltrationen und Blutungen (Abb. 4). Es erfolgte ein vorsichtiges Abgetragen der fibrinösen Membranen (Abb. 5). Intravitreal erfolgte die Eingabe von 2,2 mg Ceftazidim, 1,0 mg Vancomycin und 1,0 mg Dexamethason. Die mikrobiologische Testung der intraoperativ gewonnenen Glaskörperflüssigkeit ergab keinen Keimnachweis.

## Diagnose

Die endgültige Diagnose einer Endophthalmitis auf dem Boden einer Durchwanderungskeratitis mit perforiertem Hornhautulkus und Irisinkarzeration wurde intraoperativ gestellt.

## Diskussion

Eine Endophthalmitis ist eine seltene, schwere Infektion des Augapfels durch Bakterien oder Pilze, welche innerhalb kurzer Zeit zur irreversiblen Erblindung führen kann [5]. Bakterielle Infektionen verlaufen hochakut, mykotische Infektionen hingegen oft subakut. Exogene Endophthalmitiden werden in der Regel hervorgerufen durch Traumen, operative Eingriffe oder eine fortgeleitete Keratitis. Endogene Endophthalmitiden entstehen durch eine Septikämie [4]. Die Therapieentscheidung hängt jeweils vom klinischen Erscheinungsbild, der Dauer und der Genese ab [1, 2]. Je nach Befund kann eine Endophthalmitis innerhalb weniger Stunden zu schweren Augenschäden und zum Verlust des Sehens und des Augapfels führen. Leitsymptome sind typischerweise Visusminderung, starke Schmerzen und ein gerötetes Auge. In der Spaltlampenuntersuchung fallen zu Beginn einer Endophthalmitis ein Reizzustand der Vorderkammer mit Zellen und positivem Tyndall-Phänomen sowie eine positive Fibrinreaktion auf. Klassisch zeigen sich im Verlauf stark gemischte Injektionen, eine Reizmiosis, oft ein Hypopyon und/oder eine Vitritis [3]. Funduskopisch können neben einem zunehmend beeinträchtigten Funduseinblick Netzhautblutungen und -infiltrate beobachtet werden.

Unser Fall zeigt einen klinisch eher untypischen Verlauf einer fortgeschrittenen Endophthalmitis bei perforiertem Hornhautulkus mit fehlenden klassischen Spaltlampenbefunden wie Hypopyon, Injektion oder Reizmiosis. Ebenso verdeckten die absolute Schmerzlosigkeit sowie die plausible Anamnese bei langjährig bekannter Hornhautproblematik die Befundschwere. Eine fehlende Reizmiosis mag hier noch durch die hinteren Synechien zu erklären sein, das Fehlen einer Bindehautinjektion und insbesondere das fehlende Hypopyon bei einer offensichtlich von vorne nach hinten geleiteten Infektion darf als ungewöhnlich beurteilt werden.

Diese Besonderheit führte zu einem Verzicht auf die Durchführung eines präoperativen Ultraschalls, der natürlich bei Verdacht auf Endophthalmitis zwingend durchgeführt werden sollte. Dieser Fall zeigt daher eindringlich, wie ein bereits initial in eine bestimmte Richtung geleitetes differenzialdiagnostisches Denken den Blick auf mögliche weitere Diagnosen trüben kann. In diesem Fall wurde bei anscheinend klarer Fallkonstellation nicht ausreichend an die Möglichkeit einer schwereren Diagnose wie der Endophthalmitis gedacht. Retrospektiv war dieses nicht unbedingt entscheidend für das Endergebnis, hätte jedoch für die Operationsplanung bezüglich einer kombinierten Operation und der Operationslänge Konsequenzen gehabt. Differenzialdiagnostisch muss bei kurz zurückliegender Amnionmembranaufnähung sowie Monate zuvor stattgehabter Kataraktoperation mit Synechiolyse an eine Late-Onset-Endophthalmitis gedacht werden. Eine Late-Onset-Endophthalmitis erscheint in diesem speziellen Fall jedoch unwahrscheinlich, da in der Kontrolluntersuchung 14 Tage vor notfallmäßiger Vorstellung eine intraokulare Reizfreiheit vorlag.

Dieser Fall zeigt eindrucksvoll, dass die Durchführung eines Augenultraschalls auch bei vermeintlich eindeutigen Vorderaugenabschnittsbefunden immer erfolgen sollte, da selbst eine fulminante Endophthalmitis durchaus maskiert und untypisch verlaufen kann.

## Fazit für die Praxis

- Bei der Endophthalmitis handelt es sich um einen ophthalmologischen Notfall.
- Auch im fortgeschrittenen Stadium kann sich ein klinisch untypischer Verlauf zeigen.
- Vermeintlich eindeutige Befunde bei langjährig bekannten Patienten mit rezidivierenden Beschwerden sollten immer wieder kritisch hinterfragt werden.
- Eine Augenultraschalluntersuchung sollte bei fehlendem Einblick auf den Augenhintergrund auch bei vermeintlich eindeutigen Vorderaugenabschnittsbefunden immer erfolgen.
